# Molecular investigation of isolates from a multistate polymicrobial outbreak associated with contaminated total parenteral nutrition in Brazil

**DOI:** 10.1186/s12879-018-3287-2

**Published:** 2018-08-13

**Authors:** Marcelo Pillonetto, Lavinia Arend, Suzie M. T. Gomes, Marluce A. A. Oliveira, Loeci N. Timm, Andreza F. Martins, Afonso L. Barth, Alana Mazzetti, Lena Hersemann, Theo H. M. Smits, Marcelo T. Mira, Fabio Rezzonico

**Affiliations:** 1 Core for Advanced Molecular Investigation, Graduate Program in Health Sciences, School of Medicine, Pontifícia Universidade Católica do Paraná, Curitiba, PR Brazil; 2 Central Public Health Laboratory – State of Paraná – LACEN/PR, Molecular Bacteriology Division, São José dos Pinhais, PR Brazil; 3 Brazilian Health Surveillance Agency – ANVISA, Brasília, DF Brazil; 4 Ezequiel Dias Foundation, Central Public Health Laboratory – State of Minas Gerais, Bacteriology Division, Belo Horizonte, MG Brazil; 5 Central Public Health Laboratory – State of Rio Grande do Sul, Bacteriology Division, Porto Alegre, RS Brazil; 6 Research Laboratory on Bacterial Resistance (LABRESIS), Federal University of Rio Grande do Sul, Hospital de Clínicas, Porto Alegre, RS Brazil; 7 Environmental Genomics and Systems Biology Research Group, Institute of Natural Resource Sciences (IUNR), Zurich University of Applied Sciences ZHAW, Wädenswil, Switzerland; 8 Present Address: Scientific Computing Facility, Max Planck Institute of Molecular Cell Biology and Genetics, Pfotenhauerstrasse 108, 01307 Dresden, Germany

**Keywords:** *Acinetobacter baumannii*, *Rhizobium radiobacter*, *Phytobacter diazotrophicus*, *Pantoea*, TPN, Rep-PCR, Bacterial identification

## Abstract

**Background:**

Between November 2013 and June 2014, 56 cases of bacteremia (15 deaths) associated with the use of Total Parenteral Nutrition (TPN) and/or calcium gluconate (CG) were reported in four Brazilian states.

**Methods:**

We analyzed 73 bacterial isolates from four states: 45 from blood, 25 from TPN and three from CG, originally identified as *Acinetobacter baumannii, Rhizobium radiobacter, Pantoea* sp. or *Enterobacteriaceae* using molecular methods.

**Results:**

The first two bacterial species were confirmed while the third group of species could not be identified using standard identification protocols. These isolates were subsequently identified by Multi-Locus Sequence Analysis as *Phytobacter diazotrophicus*, a species related to strains from similar outbreaks in the United States in the 1970’s. Within each species, TPN and blood isolates proved to be clonal, whereas the *R. radiobacter* isolates retrieved from CG were found to be unrelated*.*

**Conclusion:**

This is the first report of a three-species outbreak caused by TPN contaminated with *A. baumannii*, *R. radiobacter* and *P. diazotrophicus*. The concomitant presence of clonal *A. baumannii* and *P. diazotrophicus* isolates in several TPN and blood samples, as well as the case of one patient, where all three different species were isolated simultaneously, suggest that the outbreak may be ascribed to a discrete contamination of TPN. In addition, this study highlights the clinical relevance of *P. diazotrophicus*, which has been involved in outbreaks in the past, but was often misidentified as *P. agglomerans*.

**Electronic supplementary material:**

The online version of this article (10.1186/s12879-018-3287-2) contains supplementary material, which is available to authorized users.

## Background

Total parenteral nutrition (TPN) is an important nutritional supplement for seriously ill patients, especially those incapable of oral or enteral nutrition. TPN are nutritionally rich mixtures and probably the most complex pharmaceutical dosage forms compounded by pharmacists. These infusions, composed by 50 or more constituents, including electrolytes, amino acids, dextrose, lipids and calcium gluconate (CG) [1], are also a favorable growth media for microorganisms [2]. In addition, multiple transfer steps into the same container during preparation increase the risk for microbial contamination [3]. To avoid this, strictly sterile manipulation conditions and total compliance to good manufacturing practices and quality control rules are of paramount importance [1, 4]. Adoption of these procedures helped TPN-related outbreaks to decline, but adverse events still occur worldwide, usually resulting in severe sepses with high fatality rate, especially in vulnerable individuals [5].

In nine outbreaks reviewed from 1990 to 2006, the mortality following TPN contamination was 48.9% (19/39) [5]. Incidence of bloodstream infection within patients receiving TPN has been reported between 1.3–39% [3]. Exposure to TPN is considered an independent risk factor for blood stream infections at neonate intensive care units [6]. The relative risk has been estimated as 4.69 in neonates receiving TPN through central catheter [7].

Several species of microorganisms have been involved in TPN-related outbreaks, including bacteria belonging to the *Erwinia herbicola-Enterobacter agglomerans* complex (EEC), as well as glucose non-fermenting Gram-negative bacteria, such as *Acinetobacter* spp. [8–11] and, less frequently, also *Rhizobium radiobacter* [12].

In November 2013, the municipal autorithies of the City of Curitiba, capital of the state of Paraná, southern Brazil, reported a contamination of TPN bags. The batch was immediately recalled and a retrospective statewide survey was launched, resulting in the identification of 30 cases of TPN-related bacteremia (Additional file 1: Table S1). Case definition was: patient receveing Total Parenteral Nutrition at Hospitals receiving TPN bags from the suspected producers. The finding led the Brazilian Health Surveillance Agency (ANVISA) to trigger a nation-wide epidemiological, sanitary and microbiological investigation, leading to immediate withdraw of the TPN solutions from the suspected manufacturers and closing of the involved TPN pharmaceutical industry for a three-month period. At the same time, 16 additional cases were detected in the southeastern state of Minas Gerais and were linked to a different pharmaceutical industry and one compound pharmacy based on that state, whereby the same precautions were taken by local health autorithies. Additionally, an extensive epidemiological investigation was conducted, tracing the use of the raw material used to manufacture the compound solutions in Paraná and Minas Gerais production plants. The use of the same lot of calcium gluconate in mixtures from both Paraná and Minas Gerais was investigated. The sole calcium gluconate manufacturer industrial plant in Brazil was shut down and sterility tests were launched. In March 2014, one single case of bacteremia was detected in Mogi Mirim, a municipality located southeastern state of São Paulo. From February to June 2014, further nine cases of bacteremia involving use of TPN and/or CG produced in one additional in-house pharmacy (IP) were detected in the southern state of Rio Grande do Sul (Additional file 1: Table S1).

Initial investigation, as performed by manual or automated biochemical methods at the laboratories of the hospitals where the cases were detected, resulted in the identification of mainly three species – “*P. agglomerans”*/“*Pantoea* sp.”, *A. baumannii*, and *R. radiobacter* (Additional file 1: Table S2). Alongside, there were sporadic reports of different *Enterobacteriaceae* species such as *Citrobacter amalonaticus*, *Citrobacter diversus*, *Kluyvera intermedia*, *Kluyvera* sp. and *Enterobacteriaceae* spp. Later, in June 2014, *R. radiobacter* was detected in three lots of CG by a private laboratory in Rio Grande do Sul.

Throughout this investigation, a subset of 73 bacterial isolates from blood, TPN and CG were directed to the reference Central Public Health Laboratory of Paraná – LACEN for molecular analysis. This subset was composed of all available isolates recovered during the outbreak and some TPN bags that were only partially administered to the patients. Here, we report the results of the microbiological and molecular investigation of this outbreak, with emphasis on the identification of the bacterial species involved and the tracking of the probable sources of contamination.

## Methods

### Epidemiological description of the outbreak

Demographic and epidemiological data of the cases were obtained from three different sources: epidemiological reports from municipal and state health autorithies at Paraná, Minas Gerais and Rio Grande do Sul; official reports published by ANVISA; and the Laboratory Information System at LACEN.

### Origin of isolates

Within this study, no clinical samples were handled directly, as we only obtained bacterial isolates from the involved hospitals. All isolates originated from the blood stream of case patients, from TPN bags involved in the treatment of the patients, or from CG vials. These isolates were sent to LACEN in adequate transport containers, together with the microbiological reports produced at the hospitals where the cases were ascertained. From November 2013 through June 2014, a total of 45 isolates from 27 patients were evaluated. Another 25 isolates were recovered directly from TPN (four different manufacturers from four states), while the last three came from three different lots of CG from one single compounding industry (Table 1 and Additional file 1: Table S2).

**Table 1:** Geographic and microbiological data for isolates received during the TPN outbreak in Brazil

Source of isolates	Bacteria^a^		
	EEC (Brenner XII)	*R. radiobacter*	*A. baumannii*
Blood	19 (16 pts)	9 (9 pts)	17 (11 pts)
Total parenteral nutrition	6	14	5
Calcium gluconate vials	0	3	0
Total	25	26	22
Original lab identification	*Pantoea* spp. (9)	*R. radiobacter* (18)	*A. baumannii* (16)
	*P. agglomerans* (8)	GNFGNB (7)	UNI (6)
	*C. diversus* (4)	UNI (1)	
	*C. amalonaticus* (1)		
	*K. intermedia* (1)		
	*Kluyvera* spp. (1)		
	*Enterobacteriaceae* (1)		
Geographical origin			
Paraná			
Blood	14	5	17
TPN	4	–	5
Minas Gerais			
Blood	5	–	–
TPN	2	–	–
São Paulo	–	1	–
Blood	–	–	–
TPN	–	–	–
Rio Grande do Sul			
Blood	–	3	–
TPN	–	14	–
CG	–	3	–
Hospitals Involved (cities)			
Paraná	5 (3)	4 (2)	4 (2)
Minas Gerais	5 (2)	–	–
São Paulo	–	1 (1)	–
Rio Grande do Sul	–	3 (1)	–

^a^*Abbreviations*: *Pts* Patients, *TPN* total parenteral nutrition, *CG* calcium gluconate, *GNFGNB* glucose non-fermenting Gram-negative bacteria, *UNI* unidentified

A microbiological investigation of the pharmaceutical components sterility (such as CG, vitamins, electrolytes, amino acids, and dextrose) used to produce TPN in Paraná was conducted using USP Pharmacopeia Methods (1995). In addition, different equipment parts such as bags, connectors, elastomers and others were also submitted to standard microbiological sterility tests. No microorganisms were recovered from those tests [13].

### Microbiological identification

Strains obtained in this study were routinely grown on MacConkey agar and stored at − 80 °C as glycerol stocks. Identification of all 73 isolates was performed at LACEN with the Vitek-2® platform (Mercy L’Ètoile, FR) using GN and AST-N 239 cards for species identification and antimicrobial susceptibility testing, respectively. Partial sequences (around 500 bp) of the 16S rRNA gene were obtained using the MicroSEQ 500® 16S rDNA PCR Kit (Thermo Fisher Scientific – Waltham, MA-USA) and analyzed using Le BIBI https://umr5558-bibiserv.univ-lyon1.fr/lebibi/lebibi.cgi [14] and Sepsi-Test® Blast http://www.sepsitest-blast.de/en/index.html identification tools.

### Molecular typing

**Fig. 1:**
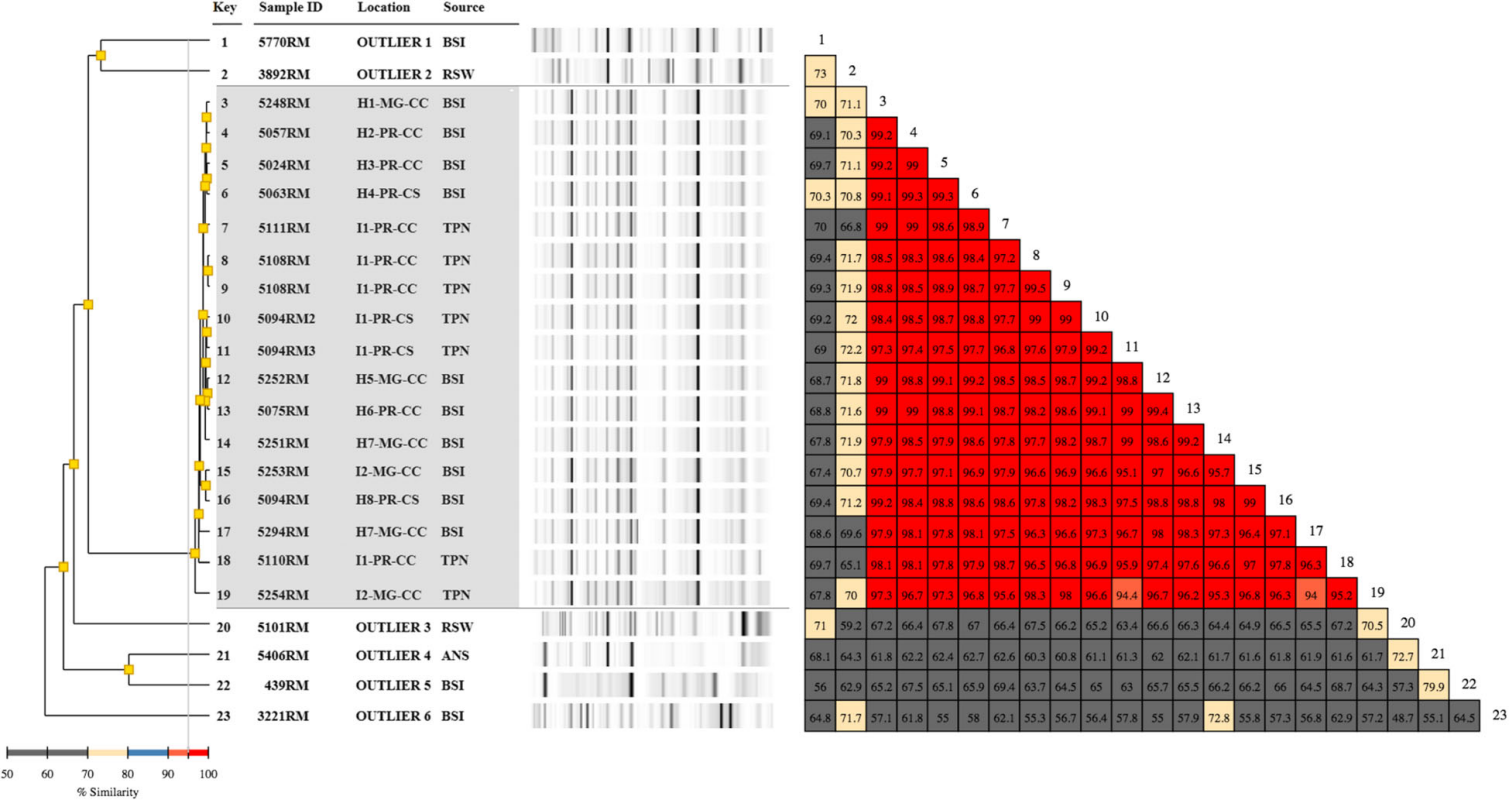
Rep-PCR based molecular typing and percentage of similarity within *Phytobacter diazotrophicus* outbreak isolates and outliers. Location: H1 to H7- hospitals numbered sequentially; I1-I2 – industries 1 and 2, respectively; MG – Minas Gerais State, PR – Paraná State; CC- capital city; CS – country-side. Source: ANS – ankle secretion; BSI – blood isolates; TPN – total parenteral nutrition; RSW – rectal swab. Shaded area: isolates recovered from the outbreak. The couloured boxes states the percentage of similarity between the two strains. The red boxes indicate higher similarity (above 95%) between strains meaning isolates are clonal. The orange boxes indicate high similarities (90–95%) meaning isolates are related –i.e: belong to the same clonal group. Yellow boxes indicate intermediate similarities (70–80%). The grey boxes indicate similarity is low (50–70%)

**Fig. 2:**
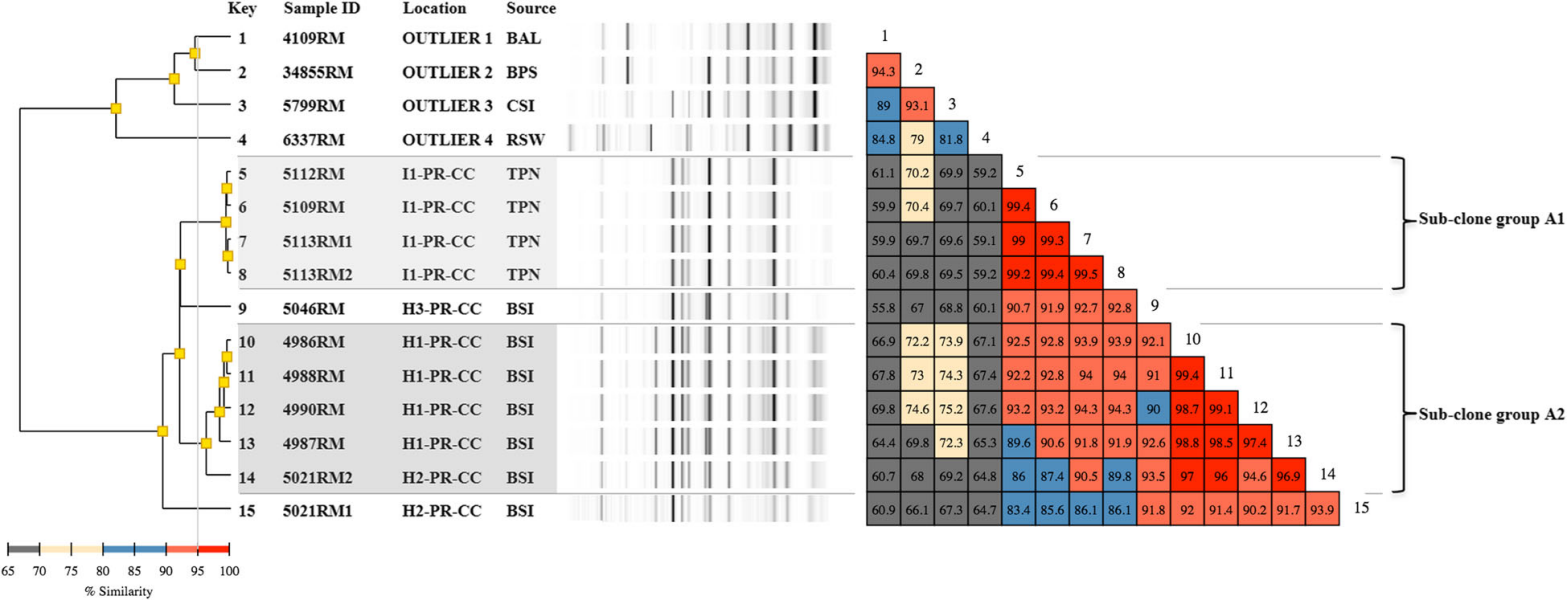
Rep-PCR based molecular typing and percentage of similarity within *Acinetobacter baumannii* outbreak isolates and outliers. Location: H1 to H3- hospitals numbered sequentially; I1-I2 – industries 1 and 2, respectively; PR – Paraná State; CC- capital city; CS – country-side. Source: BAL – bronchoalveolar lavage; BPS – Biopsy; BSI – blood isolates; CSI – Cirurgic Site Infection; TPN – total parenteral nutrition; RSW – rectal swab. Shaded area: clonal groups from the outbreak. The couloured boxes states the percentage of similarity between the two strains. The red boxes indicate higher similarity (above 95%) between strains meaning isolates are clonal. The orange boxes indicate high similarities (90–95%) meaning isolates are related –i.e: belong to the same clonal group. Yellow boxes indicate intermediate similarities (70–80%). The grey boxes indicate similarity is low (50–70%)

**Fig. 3:**
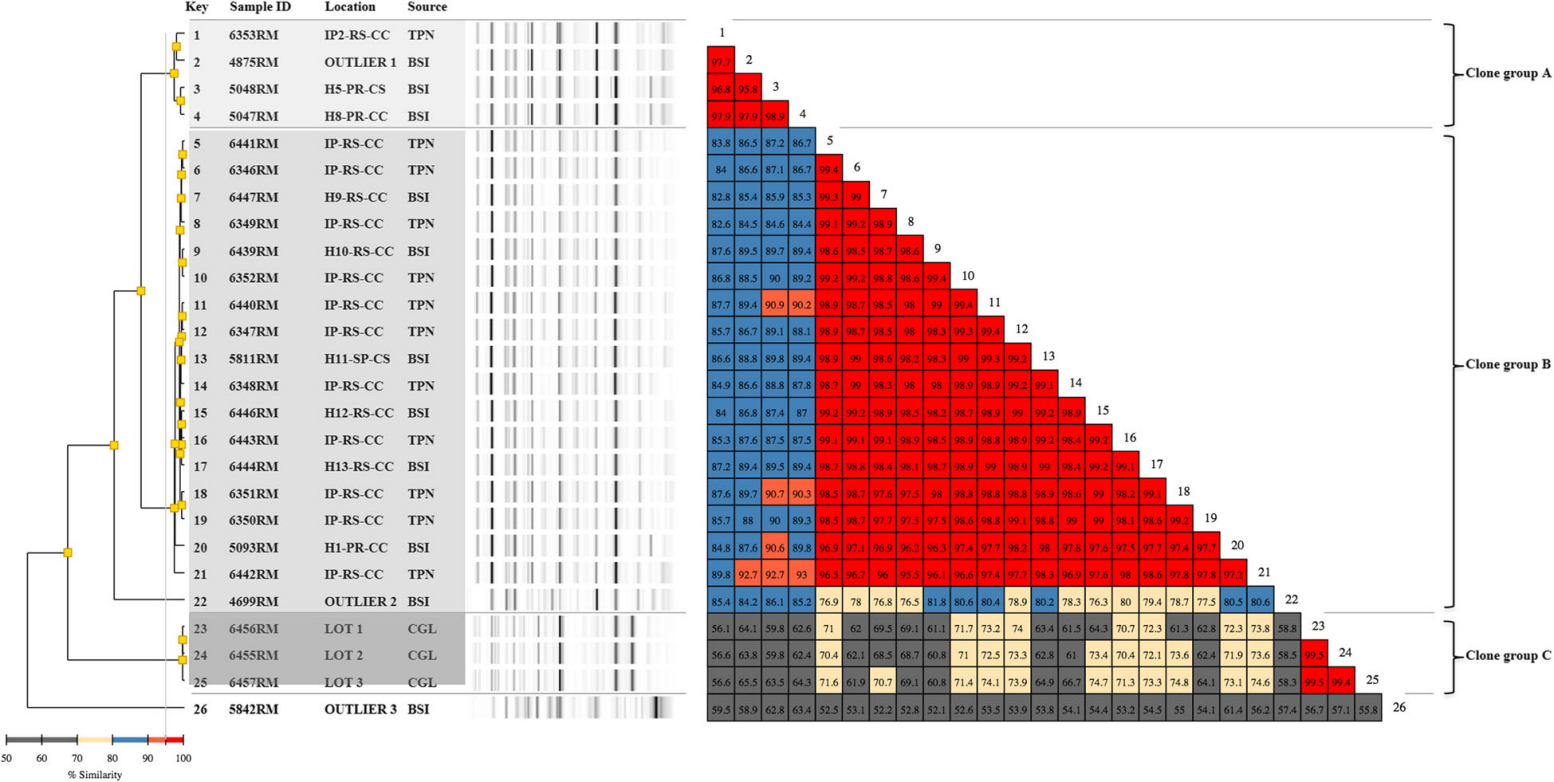
Rep-PCR based molecular typing and percentage of similarity within *Rhizobium radiobacter* outbreak isolates and outliers. Location: H1 to H13- hospitals numbered sequentially; IP-In house pharmacy; PR – Paraná State; RS – Rio Grande do Sul State; SP – São Paulo state; CC- capital city; CS – country-side. Source: BSI – blood isolates; CGL – Calcium gluconate vial; TPN – total parenteral nutrition. Shaded area: isolates belonging to the same clone or clonal group. The couloured boxes states the percentage of similarity between the two strains. The red boxes indicate higher similarity (above 95%) between strains meaning isolates are clonal. The orange boxes indicate high similarities (90–95%) meaning isolates are related –i.e: belong to the same clonal group. Yellow boxes indicate intermediate similarities (70–80%). The grey boxes indicate similarity is low (50–70%)

Molecular epidemiology analysis was performed using an automated rep-PCR system (Diversilab®, BioMerieux) as previously described [15]. Seventeen *Enterobacteriaceae* (ten from blood and seven from TPN, Fig. 1), eleven *Acinetobacter* (seven from blood and four from TPN, Fig. 2) and twenty-three *Rhizobium* (eight from blood, twelve from TPN and three from CG, Fig. 3) chosen to represent the widest diversity in isolation source and sample type were submitted to clonal analysis. Isolates sent to LACEN before or after the outbreak period (November 2013–June 2014), but from the same hospitals, if available, were included as outliers for rep-PCR profile comparision, including six “*Pantoea* spp.”, four *A. baumannii* and three *R. radiobacter* (Figs. 1, 2 and 3).

Rep-PCR results were analyzed using the Pearson correlation statistical method (Diversilab software). Isolates showing a similarity of 90% or higher were considered as related, whereas if the similarity exceeded 95%, they were assigned to the same clonal group according to Higgins et al. [16].

### Whole-genome sequencing of two Enterobacteriaceae isolates

A random isolate from TPN (5110RM) [17] and a random isolate from a blood sample (5020RM), both identified by Vitek-2® as “*Pantoea* sp.”, were selected for more detailed molecular analysis of all the outbreak isolates primarily assigned to some species within the *Enterobacteriaceae*. Whole-genome sequencing (WGS) of both isolates was performed on the Illumina MiSeq platform (Illumina Inc., San Diego, USA) and draft genomes were assembled de novo using the SeqMan NGen software included in the DNASTAR Lasergene genomics package version 12 (DNASTAR, Madison, USA) as described elsewhere [17]. Sequences of housekeeping genes *atpD*, *gyrB*, *infB* and *rpoB* were extracted from WGS data and used to perform MultiLocus Sequence Analysis (MLSA) according to the method described by Brady et al. [18]. A phylogenetic tree was constructed using the concatenated DNA sequences implementing the Neighbor-Joining method in the software MEGA 7 [19]. Average nucleotide identities (ANI) were determined from the subroutine in EDGAR 2.1 [20] after annotation of the genomes in GenDB [21].

## Results

### Epidemiological investigation

We received 45 blood isolates from 15 hospitals of seven different cities in four different states. Additionally, 25 isolates obtained from TPN administered to the patients and three isolates from CG were included in this study (Additional file 1: Table S2). Overall mortality rate was 26.8% (15/56) (Table 1 and Additional file 1: Table S1). The age distribution of the patients was as follows: 22 were below 1 year of age (medium age: 51.3 days; range: 12 to 240 days), while the remaining were older (range: 4 to 74 years) at the time blood samples were collected.

### Bacterial identification

Verification of the identity of the 73 isolates at LACEN demonstrated that three different bacterial species were present in the analyzed samples: 22 and 26 isolates were unanimously identified as *A. baumannii* complex (ABC) and *R. radiobacter*, respectively, both using the Vitek-2® platform as well as by 16S rRNA gene analyses, thus largely confirming the primary identification performed by the hospital laboratories of origin (Additional file 1: Table S2**,** accession numbers for archetypal 16S rRNA sequences: *A. baumannii* isolate 4988RM - MF403059; *R. radiobacter* isolate 5037RM - MF403063). The other 25 isolates could tentatively be assigned to the *Enterobacteriaceae* family, but were allocated to different species depending on the identification method used (see below).

The additional sterility test of pharmaceutical components in TPN production did not yield any isolates, indicating that no discrete component or equipment hardware was contaminated.

### Isolates belonging to the Enterobacteriaceae

A total of 25 *Enterobacteriaceae* isolates were referred to LACEN: 19 isolates from blood and six isolates from TPN. The blood isolates originated from 16 different patients, one of which presenting four *Enterobacteriaceae* isolates obtained on two different days. Fourteen out of the 19 *Enterobacteriaceae* blood isolates were from five hospitals in three different cities of Paraná; the remaining five isolates were from five hospitals at two different cities of Minas Gerais. Six *Enterobacteriaceae* isolates were isolated from six different lots of TPN. Four of six *Enterobacteriaceae* from TPN were from Curitiba (Paraná) and two others from Belo Horizonte (Minas Gerais).

**Fig. 4:**
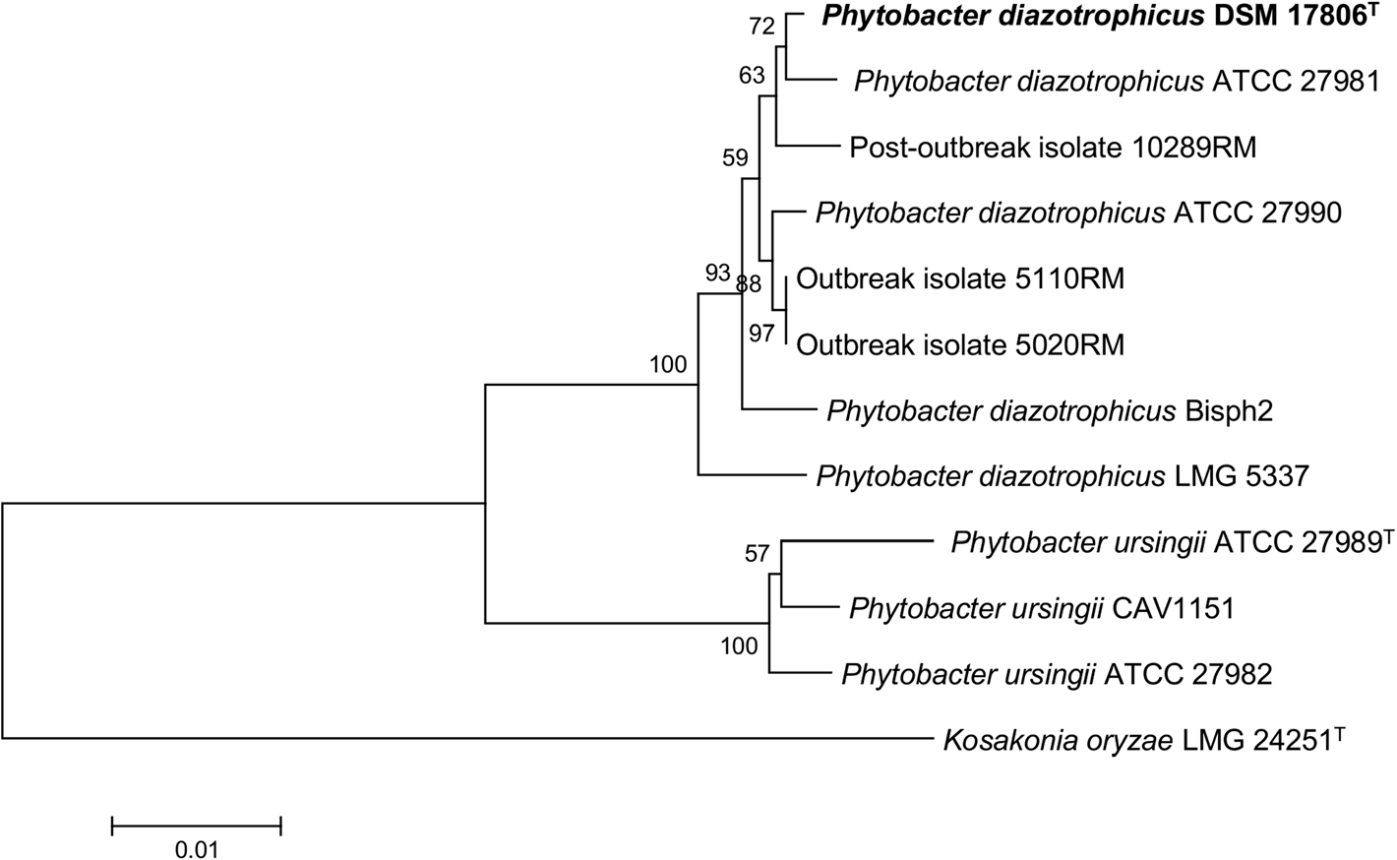
Position of outbreak isolates 5110RM and 5020RM as well as of post-outbreak isolate 10289RM within *P. diazotrophicus* as determined by MLSA using concatenated sequences of housekeeping genes *atpD*, *gyrB*, *infB* and *rpoB* (Brady et al., 2008). The tree was was inferred using the Neighbor-Joining method. Numbers at branching points are bootstrap percentage values (> 50%) based on 1000 replications. *Kosakonia oryzae* LMG 24251^T^ as used as outgroup. The scale bar represents 1% nucleotide substitutions

All *Enterobacteriaceae* isolates were identified at LACEN as *“Pantoea* sp.” using the Vitek-2 platform. On the other hand, partial 16S rRNA gene sequence analysis at the Le BIBI website identified all isolates either as ‘*Grimontella senegalensis’* or as *Phytobacter diazotrophicus*. Moreover, when the SEPSI-Test Blast website was used to check the identity of partial 16S rRNA genes, all sequences indicated *Citrobacter amalonaticus* (Additional file 1: Table S2). Manual comparison of the full 16S rRNA gene sequence of isolate 5110RM (a representative isolate from the only clonal group of *P. diazotrophicus,* obtained from TPN) against the 16S rRNA gene of the type strain *P. diazotrophicus* DSM 17806^T^ resulted in a high sequence identity (99,4%), using BLAST2N (https://blast.ncbi.nlm.nih.gov/Blast.cgi?PAGE_TYPE=BlastSearch&BLAST_SPEC=blast2seq&LINK_LOC=align2seq) [17]. This result was then checked by MLSA analysis using concatenated sequences of housekeeping genes *atpD*, *gyrB*, *infB* and *rpoB* extracted from WGS data of both strains, which confirmed the identification of the isolate as *P. diazotrophicus* [17] (Fig. 4, Additional file 1: Table S3).

Rep-PCR results for the 17 *Enterobacteriaceae* isolates analyzed showed 95% or higher similarity among them, thus confirming their clonality (Fig. 1). Since isolate 5110RM, which was previously assigned to *P. diazotrophicus* [17], is also included in this clone group, we safely can assume that all other *Enterobacteriaceae* isolates also belong to this species. Sequencing of the genome of a second clinical isolate (5020RM, obtained from blood) allowed the comparison of its MLSA genes. These were identical to those of 5110RM and displayed 99.53% similarity to type strain *P. diazotrophicus* DSM 17806^T^ [17] (Fig. 4). The genome sequence showed that the two outbreak isolates had average nucleotide identities of 99.99%, thus further supporting clonality.

Considering the isolates chosen as outliers and as well identified by Vitek-2 as ‘*Pantoea* sp.’, two of them (5770RM and 3892RM) showed around 70% similarity to the clonal outbreak isolates, while the remaining four clustered more distantly (Fig. 1). Ensuing sequence analysis of the MLSA gene set allowed their a more precise assignement of the outliers to a species, whereby only three out of six were effectively found to belong to the genus *Pantoea* and none was identified as *P. diazotrophicus* (Additional file 1: Table S4).

### *The A.* baumannii *isolates*

Twenty-two isolates of *A. baumannii* complex (ABC) were referred to LACEN, of which 17 originated from ten different patients: eight with single isolates and two with multiple isolates (four and five positive blood cultures, respectively). Five ABC isolates were originated from five different lots of TPN. All *A. baumannii* isolates were from Paraná state, and identification was confirmed both by Vitek-2 and partial 16S rRNA gene sequencing (Additional file 1: Table S2).

Four TPN isolates belonged to the same sub-clonal group (A1) with similarity above 99%; one blood isolate (5046RM) showed an average similarity of 92.0% to sub-clone group A1 (TPN) and 91.5% to sub-clone group A2 (blood isolates). Within all the latter blood isolates, similarity ranged from 94.6 to 99.4% based on rep-PCR determinations. Comparison of individual isolates between clone group A1 and A2 resulted in a similarity ranging from 86.0% (5112RM vs. 5021RM2) up to 94.3% (5113RM vs. 4990RM) (Fig. 2).

While isolates 5113RM1 and 5113RM2 obtained from the same TPN bag proved to be clonal (99.5% similarity), isolates 5021RM1 and 5021RM2 originating from the same patient failed to reach, albeit by a narrow margin (93,9%), the similarity threshold needed to be included within the same clonal group.

### *The* R. radiobacter *isolates*

Twenty-six isolates of *R. radiobacter* were referred to LACEN, nine of them originated from blood samples of nine different patients and 14 from different bags of TPN. No patient presented multiple isolates. Five out of nine isolates from blood samples were from four hospitals at two different cities in Paraná; three isolates were from three hospitals at Porto Alegre (Rio Grande do Sul) and one isolate was from Mogi Mirim (São Paulo State). Additionally, three isolates were obtained from CG vials. All isolates from TPN bags and CG vials were from Porto Alegre. Identification of all *R. radiobacter* isolates was confirmed both by Vitek-2 and partial 16S rRNA gene sequencing (Additional file 1: Table S2). Molecular typing of *R. radiobacter* strains revealed three different groups with internal similarity above 95%: clonal group A, composed of three outbreak isolates and one outlier; clonal group B with 17 outbreak isolates, and group C, with the three CG isolates. Clonal groups A and B equally contained isolates from blood and TPN and inter-group pairwise comparison of their individual isolates showed a similarity ranging from 82.6% (6353RM vs. 6349RM) to 93.0% (5047RM vs. 6442RM). It is worth to note the different clonality associated to the TPN bags from the two in-house pharmacies (IP) scrutinized, with the sole isolate analyzed from IP2 (6353RM) clustering with clonal group A, whereas all those originating from IP1 could be assigned to clonal group B. Clonal group C, composed solely of CG isolates, was even more distantly related, with a similarity to the other two groups that never exceeded 75%. Two outliers showed up as singlets (Fig. 3).

### Connections between the different organisms isolated

**Fig. 5:**
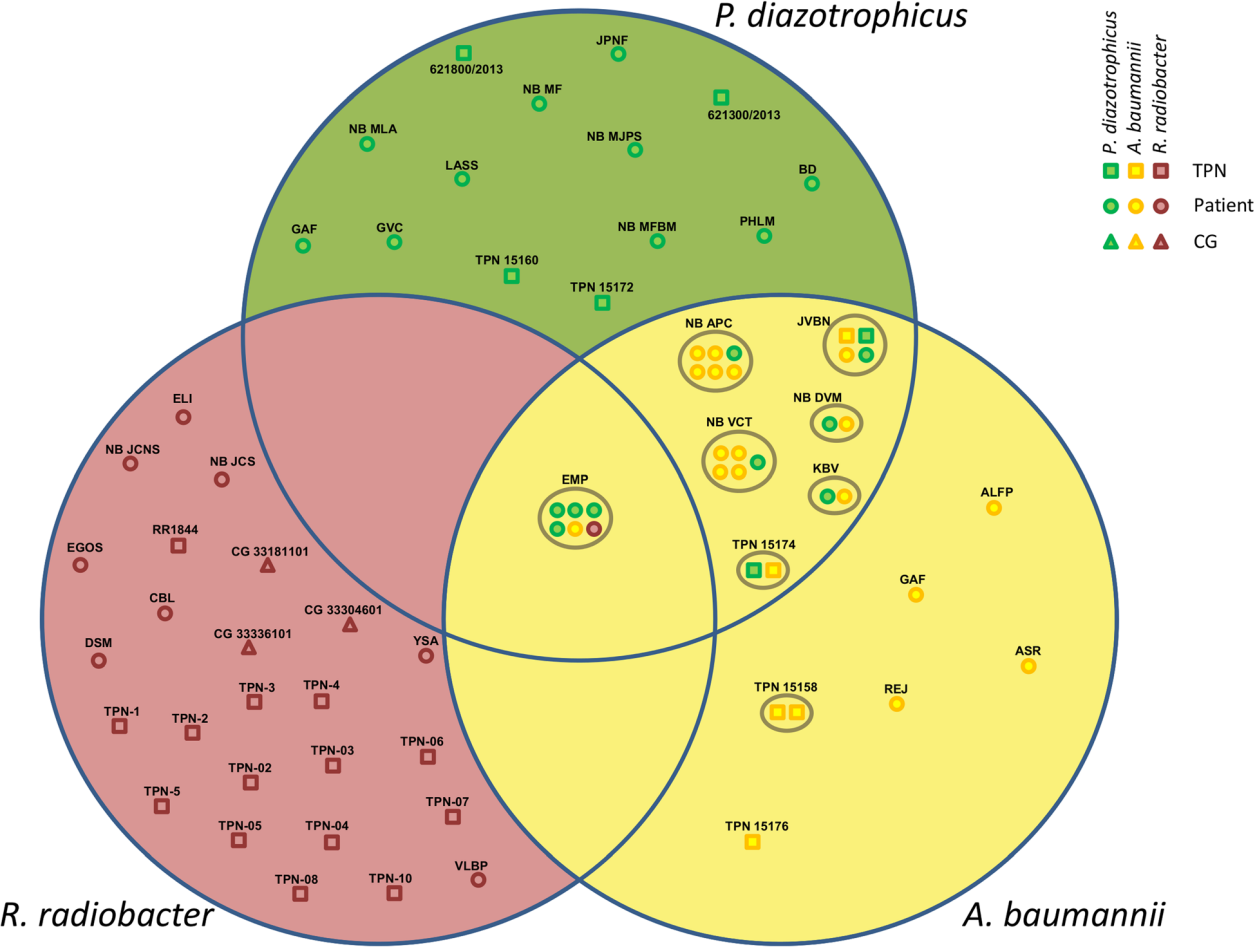
Venn diagram depicting the affiliation to the three different species involved in the outbreak of all the isolates analyzed at LACEN. Isolates obtained from the same patient, TPN bag or CG vial are regrouped by grey circles. Codes indicate individual patient or sample

*A. baumannii* and *P. diazotrophicus* were often found in mutual association both in TPN bags as well as in blood samples. Although not all *A. baumannii* isolates were tested by rep-PCR, both clonal sub-groups and one isolate (5021RM1) loosely associated to sub-group A2 could be found among those concomitant with *P. diazotrophicus*. In one case, the two species could be retrieved straight from the TPN bag administered to an infected patient (JVBN), thus reliably confirming the source of infection (Fig. 5). Blood samples from one patient in Paraná (EMP) showed an infection with all three bacteria (*A. baumannii, P. diazotrophicus* and *R. radiobacter*) simultaneously, thus hinting to the possibility of a single tribacterial outbreak. However, in no other sample were *R. radiobacter* isolates found in direct association with *A. baumannii* or *P. diazotrophicus*. *R. radiobacter* isolates of the same clonal groups found in patients in Paraná and São Paulo State were retrieved in TPN bags and blood samples from Rio Grande do Sul suggesting a connection between these events, despite the geographical distance.

## Discussion

The molecular investigation of the outbreak led to the identification of isolates belonging to three different bacterial species: *P. diazotrophicus*, *A. baumannii* and *R. radiobacter*. Molecular typing of the isolates from blood and TPN using rep-PCR revealed clonality for *P. diazotrophicus* and, to lesser extent, for *A. baumannii*. The fact that both subclonal groups of the latter species were found in association with a clonal infection by *P. diazotrophicus* hints at a single source of contamination for TPN bags (Fig. 5). Furthermore, considering that in *A. baumannii* the similarity between outbreaks isolates rarely fell considerably below 90% and was dependent on their origin (TPN or blood), the possibility that the observed differences may be due to in-run variations in the rep-PCR [22], rather than to the actual existence of different clones, cannot be discounted (Fig. 2).

On the other hand, three clonal groups could clearly be distinguished within *R. radiobacter*, with calcium gluconate isolates obviously unrelated to those originating from TPN bags and blood. This does not exclude the calcium gluconate as the possible source of contamination, but does not allow drawing any other direct conclusions. One isolate included as outlier in the study, 4875RM, noticeably clustered within clone group A (Fig. 3). Considering that 4875RM was retrieved from a hospital in Paraná in October 2013 (Additional file 1: Table S2), i.e. less than a month before the first confirmed outbreak isolates, it is possible that its existence may be indication of an earlier onset of the outbreak than originally thought.

No definitive source of contamination could be assessed beyond any doubt. The in-depth investigation by the Brazilian Health Surveillance Agency (ANVISA) targeting the various TPN components and their relative lot numbers was not conclusive. Since at least two of the three species are rather uncommon in clinical settings (*R. radiobacter* and *P. diazotrophicus*), it is possible that a concurrent, single-source contamination of TPN mixture may have occurred. This hypothesis is supported by the finding that in at least one patient, all three different species were isolated simultaneously, as well as by blood and/or TPN samples of five patients presenting both *A. baumannii* and *P. diazotrophicus* isolates (Fig. 5).

*R. radiobacter* and *P. diazotrophicus* are well known to be plant-associated, water-associated or soil-borne organisms [23, 24]. However, the reported data does not allow a definite conclusion, as calcium gluconate, one of the suspected potential sources investigated, was contaminated by a *R. radiobacter* belonging to a different clone group as the ones found in TPN or isolated from patients (Fig. 3 and Additional file 1: Table S2).

The major problem hindering a definitive identification of the origin of the outbreak is that, probably due to inter-laboratory variations in microbiological sampling procedures, not all species were regularly recovered from all cultured samples, with some laboratories only reporting the prevailing colony type. This could explain why in Paraná all three species could be identified, while in other states only *P. diazotrophicus* or *R*. *radiobacter* were isolated.

As no single origin could be identified, we cannot completely exclude the possibility that multiple sources were present, indicating a larger systematic problem with TPN. In any case, recurring outbreaks in Brazil and elsewhere in the world indicate that high standards of quality management for TPN solutions are essential. To avoid the recurrence of similar events, these would ideally include more rigid post-production controls for early detection of bacterial contaminations, including the three species identified in this study. Our study also showed that in this case sterility of the different components or equipment hardware used for TPN production was not breached, rather pointing towards the different TPN components as a source of contamination.

The identification issues within the EEC in clinical laboratories were once again exposed by this work. Indeed, at the original hospital laboratories all EEC isolates were incorrectly identified as “*Pantoea* sp.” by Vitek-2®, whereas analysis of partial 16S rRNA gene sequences using routine protocols yielded incoherent results. Only an in-depth molecular investigation implementing MLSA allowed precise identification of the involved *Enterobacteriaceae* species as *P. diazotrophicus*, an endophytic bacterium originally isolated from wild rice [17, 25] that was repeatedly involved in nosocomial outbreaks linked to the use of TPN or injectable solutions in the last five decades [26–34]. This confirms the need to adapt current clinical diagnostics protocol for an improved identification of bacteria belonging to the EEC, which is a recurring issue, especially when biochemical panels or automated systems are employed for species identification [35, 36].

## Conclusions

This study highlights the clinical relevance of *P. diazotrophicus*, a species that has been only recently described, but that was frequently misidentified as *Pantoea* sp. in the past. The complexity of this outbreak investigation, with the concomitant recurrence of three uncommon bacterial species, underpins the importance of standardized protocols for the isolation of all colony types, in order to preserve a maximum of information that is essential to reconstruct the history and origin of an epidemic. Finally, it reinforces the importance of an in-depth molecular characterization before attributing and publishing names of rare and/or atypical species involved in outbreaks in order to avoid misidentifications.

## Additional file

Additional file 1: **Table S1**. Outbreak clinical epidemiological data, including state of origin, numbers of bacteremias, mortality rate and hospitals involved. **Table S2**. Supplemental epidemiological and laboratory data, including geographical origin, sample type, original and molecular id. of all the outbreak strains sent to the LACEN reference laboratory. For each species, outliers are listed at the bottom of the page and are highlighted by the gray shading. **Table S3**. Accession numbers assigned to the sequences of the housekeeping genes used for MLSA of the strains belonging to the genus *Phytobacter*. *Kosakonia oryzae* LMG 24251^T^ was included as outgroup. **Table S4.** Accession numbers assigned to the sequences of the housekeeping genes used for the identification of the *Enterobacteriaceae* outliers included in this work. (DOCX 73 kb)

## Abbreviations

ABC: *A. baumannii* complex
ANI: Average nucleotide identity
ANVISA: Brazilian Health Surveillance Agency
CG: Calcium gluconate
EEC: *Erwinia herbicola-Enterobacter agglomerans* complex
IP: In-house pharmacy
LACEN: Central Public Health Laboratory of Paraná
MLSA: MultiLocus Sequence Analysis
TPN: Total parenteral nutrition
WGS: Whole-genome sequencing
