# Supplementary material for: Molecular investigation of isolates from a multistate polymicrobial outbreak associated with contaminated total parenteral nutrition in Brazil
Source: BMC Infect Dis. 2018 Aug 13;18:397. doi: 10.1186/s12879-018-3287-2 (PMC6090600; doi:10.1186/s12879-018-3287-2)
Supplement: Supplementary file 1 — Table S1. Outbreak clinical epidemiological data, including state of origin, numbers of bacteremias, mortality rate and hospitals involved. Table S2. Supplemental epidemiological and laboratory data, including geographical origin, sample type, original and molecular id. of all the outbreak strains sent to the LACEN reference laboratory. For each species, outliers are listed at the bottom of the page and are highlighted by the gray shading. Table S3. Accession numbers assigned to the sequences of the housekeeping genes used for MLSA of the strains belonging to the genus Phytobacter. Kosakonia oryzae LMG 24251T was included as outgroup. Table S4. Accession numbers assigned to the sequences of the housekeeping genes used for the identification of the Enterobacteriaceae outliers included in this work. (DOCX 73 kb) [file 12879_2018_3287_MOESM1_ESM.docx]

**SUPPLEMENTAL MATERIAL**

**Molecular investigation of a multistate polymicrobial outbreak associated with total parenteral nutrition in Brazil**

Marcelo Pillonetto, Lavinia Arend, Suzie M. T. Gomes, Marluce A. A. Oliveira, Loeci N. Timm, Andreza F. Martins, Afonso L. Barth, Alana Mazzetti, Lena Hersemann, Theo H.M. Smits, Marcelo T. Mira, Fabio Rezzonico

**Table S1**: Outbreak clinical epidemiological data, including state of origin, numbers of bacteremias, mortality rate and hospitals involved.

**Table S2**: Supplemental epidemiological and laboratory data, including geographical origin and clinical source, original and molecular id. of all the outbreak strains sent to the LACEN reference laboratory. For each species, outliers are listed at the bottom of the page and are highlighted by the gray shading.

**Table S3.** Accession numbers assigned to the sequences of the housekeeping genes used for MLSA of the strains belonging to the genus *Phytobacter*. *Kosakonia oryzae* LMG 24251^T^ was included as outgroup.

**Table S4.** Accession numbers assigned to the sequences of the housekeeping genes used for the identification of the *Enterobacteriaceae* outliers included in this work.

**Table S1** – Outbreak clinical epidemiological data, including state of origin, numbers of bacteremias, mortality rate and hospitals involved.

| Month/Year | State | Bacteremia Cases | Deceased (% mortality) | Hospitals Involved |
| --- | --- | --- | --- | --- |
| November 2013 | Paraná | 30 | 7 (23.3) | 6 |
| November 2013 | Minas Gerais | 16 | 2 (12.5) | 5 |
| March 2014 | São Paulo | 1 | 0 (0.0) | 1 |
| February-June 2014 | Rio Grande do Sul | 9 | 6 (66.7) | 3 |
| **TOTAL** |  | **56** | **15 (26.8%)** | **15** |

**Table S2** – Supplemental epidemiological and laboratory data, including geographical origin, sample type, original and molecular id. of all the outbreak strains sent to the LACEN reference laboratory. For each species, outliers are listed at the bottom of the page and are highlighted by the gray shading.

| **Internal Rec.** | **Sample Code** | **Origin** | **City** | **State** | **Collect.Date** | **Original Id.** | **Vitek-2 Id.** | **Vitek-2 Biotype** | **leBIBI Id.** | **SepsiTest Id.** | **Final Id.** |
| --- | --- | --- | --- | --- | --- | --- | --- | --- | --- | --- | --- |
|  |  |  |  |  |  |  |  |  | **(16S rRNA)** | **(16S rRNA)** |  |
| 4986RM | REJ | blood | CWB | PR | 08.11.2013 | *A. baumannii* | *ABC* | 0241010103500212 | *A. baumannii* | *A. baumannii* | *A. baumannii* |
| 4987RM | GAF | blood | CWB | PR | 03.11.2013 | *A. baumannii* | *ABC* | 0241010103500212 | *A. baumannii* | *A. baumannii* | *A. baumannii* |
| 4988RM | ASR | blood | CWB | PR | 31.10.2013 | *A. baumannii* | *ABC* | 0241010103500212 | *A. baumannii* | *A. baumannii* | *A. baumannii* |
| 4989RM | NB DVM | blood | CWB | PR | 29.10.2013 | *A. baumannii* | *ABC* | 0241010103500212 | *A. baumannii* | *A. baumannii* | *A. baumannii* |
| 4990RM | ALFP | blood | CWB | PR | 03.11.2013 | *A. baumannii* | *ABC* | 0241010103500212 | *A. baumannii* | *A. baumannii* | *A. baumannii* |
| 5021RM1 | NB APC | blood | CWB | PR | 31.10.2013 | *A. baumannii* | *ABC* | 0241010103500310 | *A. baumannii* | *A. baumannii* | *A. baumannii* |
| 5021RM2 | NB APC | blood | CWB | PR | 31.10.2013 | *A. baumannii* | *ABC* | 0241010103500310 | *A. baumannii* | *A. baumannii* | *A. baumannii* |
| 5021RM3 | NB APC | blood | CWB | PR | 31.10.2013 | *A. baumannii* | *ABC* | 0241010103500310 | *A. baumannii* | *A. baumannii* | *A. baumannii* |
| 5021RM4 | NB APC | blood | CWB | PR | 31.10.2013 | *A. baumannii* | *ABC* | 0241010103500310 | *A. baumannii* | *A. baumannii* | *A. baumannii* |
| 5021RM5 | NB APC | blood | CWB | PR | 31.10.2013 | *A. baumannii* | *ABC* | 0241010103500310 | *A. baumannii* | *A. baumannii* | *A. baumannii* |
| 5022RM2 | NB VCT | blood - 2 | CWB | PR | 31.10.2013 | *NI* | *ABC* | 0241010103500212 | *A. baumannii* | *A. baumannii* | *A. baumannii* |
| 5022RM3 | NB VCT | blood - 3 | CWB | PR | 31.10.2013 | *NI* | *ABC* | 0241010103500212 | *A. baumannii* | *A. baumannii* | *A. baumannii* |
| 5022RM4 | NB VCT | blood - 4 | CWB | PR | 31.10.2013 | *NI* | *ABC* | 0241010103500212 | *A. baumannii* | *A. baumannii* | *A. baumannii* |
| 5022RM5 | NB VCT | blood - 5 | CWB | PR | 31.10.2013 | *NI* | *ABC* | 0241010103500212 | *A. baumannii* | *A. baumannii* | *A. baumannii* |
| 5033RM | JVBN | TPN | CWB | PR | 14.11.2013 | *A. baumannii* | *ABC* | 0241010103500212 | *A. baumannii* | *A. baumannii* | *A. baumannii* |
| 5046RM | JVBN | blood | CWB | PR | 12.11.2013 | *A. baumannii* | *ABC* | 0241010103500212 | *A. baumannii* | *A. baumannii* | *A. baumannii* |
| 5063RM | KBV | blood | FCB | PR | 15.11.2013 | *NI* | *ABC* | 0241010103500212 | *A. baumannii* | *A. baumannii* | *A. baumannii* |
| 5094RM4 | EMP | blood-4 | PRNG | PR | 15.11.2013 | *NI* | *ABC* | 0241010103500352 | *A. baumannii* | *A. baumannii* | *A. baumannii* |
| 5109RM | TPN 15174 | TPN | CWB | PR | 05.12.2013 | *A. baumannii* | *ABC* | 0241010303500302 | *A. baumannii* | *A. baumannii* | *A. baumannii* |
| 5112RM | TPN 15176 | TPN | CWB | PR | 05.12.2013 | *A. baumannii* | *ABC* | 0241010303500210 | *A. baumannii* | *A. baumannii* | *A. baumannii* |
| 5113RM1 | TPN 15158 | TPN | CWB | PR | 05.12.2013 | *A. baumannii* | *ABC* | 0241010303500300 | *A. baumannii* | *A. baumannii* | *A. baumannii* |
| 5113RM2 | TPN 15158 | TPN | CWB | PR | 05.12.2013 | *A. baumannii* | *ABC* | 0241010303500310 | *A. baumannii* | *A. baumannii* | *A. baumannii* |
| 3485RM | PHZ | BPS | CWB | PR | 26.11.2012 | *A. baumannii* | ABC | 0241010103500210 | *A. baumannii* | *A. baumannii* | *A. baumannii* |
| 4109RM | SJGA | BAL | PRNG | PR | 06.05.2013 | *A. baumannii* | *ABC* | 0241010103500210 | *A. baumannii* | *A. baumannii* | *A. baumannii* |
| 5799RM | JPS | CSI | CWB | PR | 26.03.2014 | *A. baumannii* | *ABC* | 0241010103500300 | *A. baumannii* | *A. baumannii* | *A. baumannii* |
| 6337RM | RS | RSW | CWB | PR | 11.06.2014 | *A. baumannii* | *ABC* | 0241010103500350 | *A. baumannii* | *A. baumannii* | *A. baumannii* |

| **Internal Rec.** | **Sample Code** | **Origin** | **City** | **State** | **Collect.Date** | **Original Id.** | **Vitek-2 Id.** | **Vitek-2 Biotype** | **leBIBI Id.** | **SepsiTest Id.** | **Final Id.** |
| --- | --- | --- | --- | --- | --- | --- | --- | --- | --- | --- | --- |
|  |  |  |  |  |  |  |  |  | **(16S rRNA)** | **(16S rRNA)** |  |
| 5020RM | NB MFBM | blood | CWB | PR | 31.10.2013 | *Pantoea sp.* | *Pantoea spp.* | 0607734557500050 | *G. senegalensis* | *C. amalonaticus* | *P. diazotrophicus* |
| **5022RM1** | **NB VCT** | blood - 1 | CWB | PR | 31.10.2013 | *Pantoea sp.* | *Pantoea spp.* | 0607734557500050 | *G. senegalensis* | *C. amalonaticus* | *P. diazotrophicus* |
| 5024RM | BD | blood | CWB | PR | 14.11.2013 | *P. agglomerans* | *Pantoea spp.* | 0607734557500050 | *G. senegalensis* | *C. amalonaticus* | *P. diazotrophicus* |
| 5025RM | GAF | blood | CWB | PR | 11.11.2013 | *P. agglomerans* | *Pantoea spp.* | 0607734557500050 | *G. senegalensis* | *C. amalonaticus* | *P. diazotrophicus* |
| 5026RM | NB DVM | blood | CWB | PR | 11.11.2013 | *P. agglomerans* | *Pantoea spp.* | 0607734557500050 | *G. senegalensis* | *C. amalonaticus* | *P. diazotrophicus* |
| 5027RM | GVC | blood | CWB | PR | 11.11.2013 | *P. agglomerans* | *Pantoea spp.* | 0607734557500050 | *G. senegalensis* | *C. amalonaticus* | *P. diazotrophicus* |
| 5032RM | JVBN | TPN | CWB | PR | 14.11.2013 | *C. amalonaticus* | *Pantoea spp.* | 0607734557500050 | *G. senegalensis* | *C. amalonaticus* | *P. diazotrophicus* |
| 5034RM | JVBN | blood | CWB | PR | 12.11.2013 | *P. agglomerans* | *Pantoea spp.* | 0607734557500050 | *G. senegalensis* | *C. amalonaticus* | *P. diazotrophicus* |
| 5036RM | PHLM | blood | CWB | PR | 14.11.2013 | *Kluyvera sp.* | *Pantoea spp.* | 0607734557500050 | *G. senegalensis* | *C. amalonaticus* | *P. diazotrophicus* |
| 5057RM | NB APC | blood | CWB | PR | 19.11.2013 | *Pantoea sp.* | *Pantoea spp.* | 0607734557500050 | *G. senegalensis* | *C. amalonaticus* | *P. diazotrophicus* |
| 5063RM | KBV | blood | FCB | PR | 15.11.2013 | *Enterobact.* | *Pantoea spp.* | 0607734557500050 | *G. senegalensis* | *C. amalonaticus* | *P. diazotrophicus* |
| 5075RM | EMP | blood | PRNG | PR | 13.11.2013 | *C. diversus* | *Pantoea spp.* | 0607734557500050 | *G. senegalensis* | *C. amalonaticus* | *P. diazotrophicus* |
| 5094RM1 | EMP | blood-1 | PRNG | PR | 15.11.2013 | *C. diversus* | *Pantoea spp.* | 0607734557500050 | *G. senegalensis* | *C. amalonaticus* | *P. diazotrophicus* |
| 5094RM2 | EMP | blood-2 | PRNG | PR | 15.11.2013 | *C. diversus* | *Pantoea spp.* | 0607734557500050 | *G. senegalensis* | *C. amalonaticus* | *P. diazotrophicus* |
| 5094RM3 | EMP | blood-3 | PRNG | PR | 15.11.2013 | *C. diversus* | *Pantoea spp.* | 0607734557500050 | *G. senegalensis* | *C. amalonaticus* | *P. diazotrophicus* |
| 5108RM | TPN 15174 | TPN | CWB | PR | 05.12.2013 | *E. agglomerans* | *Pantoea spp.* | 0607734557500050 | *G. senegalensis* | *C. amalonaticus* | *P. diazotrophicus* |
| 5110RM | TPN 15160 | TPN | CWB | PR | 05.12.2013 | *E. agglomerans* | *Pantoea spp.* | 0607734557500050 | *G. senegalensis* | *C. amalonaticus* | *P. diazotrophicus* |
| 5111RM | TPN 15172 | TPN | CWB | PR | 05.12.2013 | *E. agglomerans* | *Pantoea spp.* | 0607734557500050 | *G. senegalensis* | *C. amalonaticus* | *P. diazotrophicus* |
| 5248RM | NB MLA | blood | BH | MG | 13.11.2013 | *K. intermedia* | *Pantoea spp.* | 0607734557500050 | *G. senegalensis* | *C. amalonaticus* | *P. diazotrophicus* |
| 5249RM | NB MF | blood | BH | MG | 06.11.2013 | *Pantoea sp.* | *Pantoea spp.* | 0607734557500050 | *G. senegalensis* | *C. amalonaticus* | *P. diazotrophicus* |
| 5250RM | LASS | blood | UBL | MG | 13.11.2013 | *Pantoea sp.* | *Pantoea spp.* | 0607734557500050 | *G. senegalensis* | *C. amalonaticus* | *P. diazotrophicus* |
| 5251RM | JPNF | blood | BH | MG | 16.11.2013 | *Pantoea sp.* | *Pantoea spp.* | 0607734557500050 | *G. senegalensis* | *C. amalonaticus* | *P. diazotrophicus* |
| 5252RM | NB MJPS | blood | BH | MG | 30.09.2013 | *Pantoea sp.* | *Pantoea spp.* | 0607734557500050 | *G. senegalensis* | *C. amalonaticus* | *P. diazotrophicus* |
| 5253RM | 621800/2013 | TPN | BH | MG | 19.11.2013 | *Pantoea sp.* | *Pantoea spp.* | 0607734557500050 | *G. senegalensis* | *C. amalonaticus* | *P. diazotrophicus* |
| 5254RM | 621300/2013 | TPN | BH | MG | 20.11.2013 | *Pantoea sp.* | *Pantoea spp.* | 0607734557500050 | *G. senegalensis* | *C. amalonaticus* | *P. diazotrophicus* |
| 439RM | AFF | blood | CWB | PR | 30.10.2011 | *Pantoea sp.* | *Pantoea* spp. | 4605710051540210 | NA | NA | *P. brenneri* |
| 3221RM | ECA | blood | CWB | PR | 25.09.2012 | *Pantoea sp.* | *Pantoea* spp. | 4407610153540210 | NA | NA | *P. septica* |
| 3892RM | NB PRP | RSW | CWB | PR | 02.04.2013 | *Pantoea sp.* | *Pantoea* spp. | 4607714573560010 | NA | NA | *K. pneumoniae* |
| 5101RM | SMSK | RSW | CWB | PR | 31.10.2013 | *Pantoea sp.* | *Pantoea* spp. | NA | NA | NA | *C. youngae* |
| 5406RM | CM | ANS | CWB | PR | 17.02.2014 | *Pantoea sp.* | *Pantoea* spp. | 4005510052540210 | NA | NA | *P. vagans* |
| 5770RM | NNMP | blood | CWB | PR | 17.03.2014 | *P. agglomerans* | *Pantoea* spp. | 4225730441510210 | *G. senegalensis* | *C. amalonaticus* | *P. calida* |

| **Internal Rec.** | **Sample Code** | **Origin** | **City** | **State** | **Collect.Date** | **Original Id.** | **Vitek-2 Id.** | **Vitek-2 Biotype** | **leBIBI Id.** | **SepsiTest Id.** | **Final Id.** |
| --- | --- | --- | --- | --- | --- | --- | --- | --- | --- | --- | --- |
|  |  |  |  |  |  |  |  |  | **(16S rRNA)** | **(16S rRNA)** |  |
| 5035RM | NB JCS | blood | CWB | PR | 11.11.2013 | *GNFGNB* | *R. radiobacter* | 6520100300200000 | *R. radiobacter* | *R. radiobacter* | *R. radiobacter* |
| 5037RM | ELI | blood | CWB | PR | 12.11.2013 | *GNFGNB* | *R. radiobacter* | 6520100300200000 | *R. radiobacter* | *R. radiobacter* | *R. radiobacter* |
| 5047RM | YSA | blood | CWB | PR | 11.11.2013 | *GNFGNB* | *R. radiobacter* | 2720104200200000 | *R. radiobacter* | *R. radiobacter* | *R. radiobacter* |
| 5048RM | EMP | blood-5 | PRNG | PR | 15.11.2013 | *NI* | *R. radiobacter* | 6720304350600000 | *R. radiobacter* | *R. radiobacter* | *R. radiobacter* |
| 5093RM | NB JCNS | blood | CWB | PR | 29.11.2013 | *GNFGNB* | *R. radiobacter* | 6720300310600000 | *R. radiobacter* | *R. radiobacter* | *R. radiobacter* |
| 5811RM | EGOS | blood | MM | SP | 13.03.2014 | *R. radiobacter* | *R. radiobacter* | 6520100300200000 | *R. radiobacter* | *R. radiobacter* | *R. radiobacter* |
| 6346RM | TPN-04 | TPN | POA | RS | 09.05.2014 | *R. radiobacter* | *R. radiobacter* | 6720325750621020 | *R. radiobacter* | *R. radiobacter* | *R. radiobacter* |
| 6347RM | TPN-05 | TPN | POA | RS | 09.05.2014 | *R. radiobacter* | *R. radiobacter* | 6520100300200000 | *R. radiobacter* | *R. radiobacter* | *R. radiobacter* |
| 6348RM | TPN-03 | TPN | POA | RS | 09.05.2014 | *R. radiobacter* | *R. radiobacter* | 6520100300200000 | *R. radiobacter* | *R. radiobacter* | *R. radiobacter* |
| 6349RM | TPN-02 | TPN | POA | RS | 09.05.2014 | *R. radiobacter* | *R. radiobacter* | 6520100300200020 | *R. radiobacter* | *R. radiobacter* | *R. radiobacter* |
| 6350RM | TPN-07 | TPN | POA | RS | 09.05.2014 | *R. radiobacter* | *R. radiobacter* | 6520100300200000 | *R. radiobacter* | *R. radiobacter* | *R. radiobacter* |
| 6351RM | TPN-08 | TPN | POA | RS | 09.05.2014 | *R. radiobacter* | *R. radiobacter* | 6520100300200000 | *R. radiobacter* | *R. radiobacter* | *R. radiobacter* |
| 6352RM | TPN-10 | TPN | POA | RS | 09.05.2014 | *R. radiobacter* | *R. radiobacter* | 6520100300200000 | *R. radiobacter* | *R. radiobacter* | *R. radiobacter* |
| 6353RM | TPN-06 | TPN | POA | RS | 09.05.2014 | *R. radiobacter* | *R. radiobacter* | 6520100300200000 | *R. radiobacter* | *R. radiobacter* | *R. radiobacter* |
| 6439RM | RR1844 | TPN | POA | RS | 09.05.2014 | *R. radiobacter* | *R. radiobacter* | 6520100300200000 | *R. radiobacter* | *R. radiobacter* | *R. radiobacter* |
| 6440RM | TPN- 1 | TPN | POA | RS | 09.05.2014 | *R. radiobacter* | *R. radiobacter* | 6520100300200000 | *R. radiobacter* | *R. radiobacter* | *R. radiobacter* |
| 6441RM | TPN- 2 | TPN | POA | RS | 09.05.2014 | *R. radiobacter* | *R. radiobacter* | 6520100300200000 | *R. radiobacter* | *R. radiobacter* | *R. radiobacter* |
| 6442RM | TPN- 3 | TPN | POA | RS | 09.05.2014 | *R. radiobacter* | *R. radiobacter* | 6520100300200000 | *R. radiobacter* | *R. radiobacter* | *R. radiobacter* |
| 6443RM | TPN- 4 | TPN | POA | RS | 09.05.2014 | *R. radiobacter* | *R. radiobacter* | 6520100300200000 | *R. radiobacter* | *R. radiobacter* | *R. radiobacter* |
| 6444RM | TPN- 5 | TPN | POA | RS | 09.05.2014 | *R. radiobacter* | *R. radiobacter* | 6520100300200000 | *R. radiobacter* | *R. radiobacter* | *R. radiobacter* |
| 6445RM | VLBP | blood | POA | RS | NI | *R. radiobacter* | *R. radiobacter* | 6520100300200000 | *R. radiobacter* | *R. radiobacter* | *R. radiobacter* |
| 6446RM | DSM | blood | POA | RS | NI | *R. radiobacter* | *R. radiobacter* | 6520100310200000 | *R. radiobacter* | *R. radiobacter* | *R. radiobacter* |
| 6447RM | CBL | blood | POA | RS | NI | *R. radiobacter* | *R. radiobacter* | 6520100300200000 | *R. radiobacter* | *R. radiobacter* | *R. radiobacter* |
| 6455RM | CG 33304601 | CG | POA | RS | NI | *GPB* | *R. radiobacter* | 6520101300200000 | *R. radiobacter* | *R. radiobacter* | *R. radiobacter* |
| 6456RM | CG 33336101 | CG | POA | RS | NI | *GPB* | *R. radiobacter* | 6520105350200000 | *R. radiobacter* | *R. radiobacter* | *R. radiobacter* |
| 6457RM | CG 33181101 | CG | POA | RS | NI | *GPB* | *R. radiobacter* | 6520101310200000 | *R. radiobacter* | *R. radiobacter* | *R. radiobacter* |
| 4699RM | YDMO | blood | CWB | PR | 01.09.2013 | *R. radiobacter* | *R. radiobacter* | 6120101300220000 | *R. radiobacter* | *R. radiobacter* | *R. radiobacter* |
| 4875RM | GPFW | blood | CWB | PR | 14.10.2013 | *R. radiobacter* | *R. radiobacter* | 6520100300200000 | *R. radiobacter* | *R. radiobacter* | *R. radiobacter* |
| 5842RM | VMS | blood | CWB | PR | 25.03.2013 | GNFGNB | *R. radiobacter* | 6020101311060000 | *R. radiobacter* | *R. radiobacter* | *R. radiobacter* |

Internal Rec – internal record number; Collect. date – sample collection date; Original Id. – original laboratory identification; Vitek-2 Id. – identification by GNI card (Vitek-2, bioMerieux); Id. partial 16S leBIBI – identification using Microseq partial 16SrDNA sequencing (Thermo Fisher Scientific) with leBIBI analysis; Id. partial 16S SepsiTest – identification using Microseq partial 16S rDNA sequencing (Thermo Fisher Scientific) with SepsiTest analysis; Final Id – definitive identification; NA – Not available; NI – Not informed; D – days; M – months; Y – year; CG – calcium gluconate; ANS – ankle secretion; RSW – rectal swab; TPN – total parenteral nutrition; CWB – Curitiba; FCB – Francisco Beltrão; MM – Mogi Mirim; POA – Porto Alegre; PRNG – Paranaguá; MG – Minas Gerais; PR – Paraná; RS – Rio Grande do Sul; SP – São Paulo.

| **Table S3.** Accession numbers assigned to the sequences of the housekeeping genes used for MLSA of the strains belonging to the genus *Phytobacter*. *Kosakonia oryzae* LMG 24251^T^ was included as outgroup. | | | | | |
| --- | --- | --- | --- | --- | --- |
| **Species** | **GenBank accession numbers** | | | | |
| Strains |  | *atpD* | *gyrB* | *infB* | *rpoB* |
| ***Kosakonia oryzae*** |  |  |  |  |  |
| LMG 24251^T^ |  | JX424886 | JX425016 | JX425145 | JX425272 |
| ***Phytobacter diazotrophicus*** |  |  |  |  |  |
| 5110RM |  | KY296460 | KY296473 | KY296476 | KY296489 |
| 5020RM |  | MF409221 | MF409228 | MF409235 | MF409216 |
| 10289RM |  | KY296459 | KY296472 | KY296475 | KY296488 |
| ATCC 27981 |  | KY296462 | FJ617357 | KY296478 | KY296491 |
| ATCC 27990 |  | KY296467 | FJ617358 | KY296483 | KY296496 |
| Bisph2 |  | NZ_JXAF00000000 | NZ_JXAF00000000 | NZ_JXAF00000000 | NZ_JXAF00000000 |
| DSM 17806^T^ |  | KY296461 | KY296474 | KY296477 | KY296490 |
| LMG 5337 |  | KY296469 | FJ617359 | KY296485 | KY296498 |
| ***Phytobacter ursingii*** |  |  |  |  |  |
| ATCC 27982 |  | KY296463 | FJ617360 | KY296479 | KY296492 |
| ATCC 27989^T^ |  | KY296466 | FJ617361 | KY296482 | KY296495 |
| CAV1151 |  | NZ_CP011602 | NZ_CP011602 | NZ_CP011602 | NZ_CP011602 |

| **Table S4.** Accession numbers assigned to the sequences of the housekeeping genes used for the identification of the *Enterobacteriaceae* outliers included in this work. | | | | | | | |
| --- | --- | --- | --- | --- | --- | --- | --- |
| **Isolate number** | **GenBank accession numbers** | | | | | | **Final identification** |
|  |  | *atpD* | *gyrB* | *infB* | *rpoB* |  |  |
| 439RM |  | MF409218 | MF409225 | MF409232 | MF409213 |  | *Pantoea brenneri* |
| 3221RM |  | MF409219 | MF409226 | MF409233 | MF409214 |  | *Pantoea septica* |
| 3892RM |  | MF409220 | MF409227 | MF409234 | MF409215 |  | *Klebsiella pneumoniae* |
| 5101RM |  | MF409222 | MF409229 | MF409236 | n.a. |  | *Citrobacter youngae* |
| 5406RM |  | MF409223 | MF409230 | MF409237 | n.a. |  | *Pantoea vagans* |
| 5770RM |  | MF409224 | MF409231 | MF409238 | MF409217 |  | *Pantoea calida* |
